# Engineering drive–selection balance for localized population suppression with neutral dynamics

**DOI:** 10.1073/pnas.2414207122

**Published:** 2025-02-04

**Authors:** Katie Willis, Austin Burt

**Affiliations:** ^a^Department of Life Sciences, Faculty of Natural Sciences, Imperial College London, Silwood Park, Ascot SL57PY, United Kingdom

**Keywords:** genetic biocontrol, population suppression, drive—selection balance, population genetic engineering

## Abstract

While the release of sterile males has been highly successful in suppressing some pest populations, it is impractical for many species due to the males disappearing after a single generation, necessitating large, repeated releases to maintain sufficient impact. Synthetic gene drives promise more efficient approaches since they can increase in frequency from rare, yet this also allows them to spread across a landscape, which may not always be desired. Between these two extremes are selectively neutral genetic constructs which persist at the frequency they are released, offering the potential for efficient suppression that remains localized. One way to achieve this would be to have perfect balance, at all construct frequencies, between gene drive increasing frequency and selection decreasing it. Here, we describe a way to closely approximate this balance using a toxin–antidote genetic construct that causes recessive lethality or sterility, encodes a genomic editor that makes dominant lethal or sterile edits in the genome, and provides protection against the action or consequences of the editing. Computer modeling shows that this design can be 100-fold more efficient than sterile males, increasing to 1,000-fold when released alongside a genetic booster. We describe designs for CRISPR-based molecular construction, including options that avoid using recoded genes as antidotes.

## Significance

Genetic biocontrol has the potential to be a highly effective, species-specific, and environmentally friendly tool for controlling pest populations. Here, we propose a strategy that combines high efficiency (up to 100-fold greater than current alternatives such as the release of sterile males) while still allowing targeted control of specific populations. This high efficiency is achieved by introducing a genetic element causing the death or sterility of individuals carrying two copies of the gene and of the progeny of heterozygous carriers not inheriting the gene. As a result, the natural selection against the gene is largely canceled out by its preferential transmission. We also propose several ways such constructs could be designed using currently available gene editing tools.

Pest populations continue to impose a substantial health and economic burden on humanity by transmitting vector-borne diseases, harming crops, and causing unwanted environmental change. Recent advancements in genome and molecular engineering technologies have facilitated the development of a variety of genetic approaches for controlling pest populations. These involve releasing modified members of the pest species designed to mate with those in the wild and reduce harm (1, 2). One approach is for the released individuals to suppress the density of the population by interfering with its ability to successfully reproduce, thereby reducing the overall harm caused. Of these, the most widely used strategy is the sterile insect technique (SIT), where the released males are sterilized through irradiation (3), or related techniques where males are effectively sterilized by *Wolbachia* cytoplasmic incompatibility (4–6) or genetic modification (7, 8). These approaches have been successful in suppressing some populations (9–11), but significant and sustained suppression requires repeat, inundative releases, making it impractical for large target populations or for species difficult to rear in the laboratory.

One reason for the inefficiency of sterile male releases is that the causal agent does not persist from generation to generation, and thus, repeated releases are required to maintain impact. The application of selfish genetic elements that spread from low frequency (gene drive) has been proposed as one way to increase efficiency by reducing the numbers one needs to release (1, 12, 13). Here, smaller releases are required because the genetic construct not only can persist from generation to generation but moreover can increase in frequency and spread to neighboring populations wherever there is gene flow. While this may be desirable in species that are harmful everywhere they exist, it may not always be appropriate if control is only desirable in part of the species range. In these latter cases, it would be useful if the genetic element responsible for the reproductive load persisted over multiple generations, requiring smaller releases, but did not increase in frequency, preventing spread far from the release site. If the genetic construct persisted at the frequency it was released at, being neither selected for or against, while nevertheless imposing a reproductive load upon the population, the result would be a selectively neutral population suppressor (14).

In this paper, we propose and model one way to achieve this by pairing a load-inducing construct with a toxin–antidote drive mechanism that, under idealized conditions, perfectly counteracts the negative selection. We compare the efficiency of our design with alternate strategies for localizable suppression, assess its robustness to molecular imperfections, and describe how it could be constructed by combining existing well-known molecular tools. This work presents the initial step (theoretical exploration) of a typical genetic biocontrol developmental pipeline for a construct of this kind and stimulates progression to the next stages: developing the approach in organisms of interest within the laboratory and evaluating their impact in the field.

## 1. Results

### 1.1. Engineering Drive–Selection Balance.

One conceptually simple way to reduce the number of individuals in a population is to introduce a genetic modification that reduces individual fitness. The most robust way to do this is to disrupt a gene which is needed for survival or reproduction, and disruption of most essential genes results in recessive fitness effects. Alleles with reduced fitness would usually decline in frequency due to natural selection, and for a fully recessive allele, the strength of selection would be proportional to its frequency, being very small when rare, since the allele is most often in heterozygotes where there are no fitness costs, and large when common, because the allele is more likely to be found in the homozygous state where it does not survive. One way to counteract this selection would be to combine the recessive lethal allele with a gene drive component that perfectly balances the negative selection. A homing gene drive would be too strong, particularly when the gene is rare, making the combination more suited as a low-threshold suppression strategy designed to spread across a landscape (15–19). Rather, to perfectly balance a recessive lethal mutation, drive must be very weak at low frequency, increasing in strength in proportion to allele frequency. One class of gene drive mechanisms that can have this property are those based on toxin–antidote interactions (13, 20, 21). The most promising and easiest to engineer are cleave and rescue systems that use CRISPR/Cas9 technology, in which Cas9 “cleaves” a target gene and creates an edited nonfunctional version which acts as a toxin and the antidote is a recoded version of the target gene that “rescues” function but is resistant to Cas9 cleavage (22, 23).

**Fig. 1.**
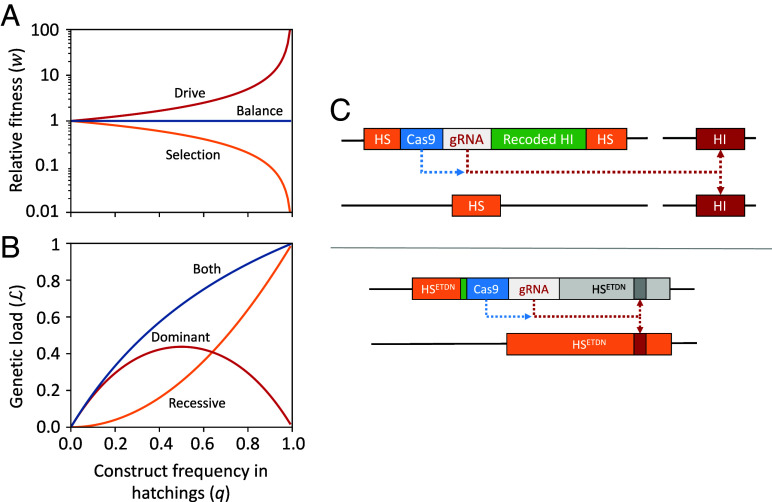
Population genetic modeling to demonstrate balance between drive and selection and possible molecular configurations of the proposed construct. (*A*) The relative fitness (w) of different genetic constructs as a function of their frequency (q) in a population at Hardy–Weinberg equilibrium. Fitnesses are calculated from changes in construct frequency over a single generation, censused at the hatching stage: $w=\left(\frac{q_{t+1}}{1-q_{t+1}}\right)/\left(\frac{q_{t}}{1-q_{t}}\right)$, where t is the generation. The relative fitness of an allele which causes recessive lethality (orange) is comparable to wild type at low frequency and decreases as its frequency increases (w=1-q). In contrast, while a cleave-and-rescue-based drive allele which creates dominant lethal mutations and provides protection against them also exhibits fitness comparable to the WT at very low frequency, its fitness increases with frequency due to drive ($w=\frac{1}{1-q}$; red). When combined (blue), the fitness advantage of the drive perfectly counteracts the fitness costs of the recessive lethal allele, resulting in fitness equal to the wild type at all frequencies (w=1). (*B*) The reproductive load (L) imposed on the population by an allele which causes recessive lethality (orange), a cleave-and-rescue-based drive allele which creates dominant lethal mutations and provides protection against them (red), or an allele with both features (blue) as a function of allele frequency. Load is calculated as the proportional reduction in reproductive output compared to a wild-type population over a single generation. (*C*) Possible CRISPR/Cas9 molecular configurations. Recessive lethality can be achieved by inserting the construct into a haplosufficient gene (HS, orange box) required for survival in both sexes such that it disrupts function. The *Upper* panel illustrates how the cleave and rescue drive can be implemented using two genes, in which the genetic construct encodes a Cas9 and gRNA which generates nonfunctional edits in a haploinsufficient gene (HI, red box) during gametogenesis. Protection can be achieved by incorporating a recoded version of the HI gene into the construct that cannot be edited by the gRNA but is still able to fully function (Recoded HI, green box). The *Lower* panel shows an alternative implementation involving a single HS gene that contains one or more sites that are editable to a dominant negative (ETDN) by the genomic editor (HS^ETDN^, orange box). If the construct is inserted upstream of the edit and generates a premature stop codon (green box) that prevents the dominant-negative allele from being expressed the construct provides protection against edits in cis, without the need for a recoded copy of the gene. Note that although we have used CRISPR/Cas9 for illustration, alternative engineerable genome editors would also be suitable.

To test whether a cleave and rescue drive would balance selection against a recessive allele, we constructed an analytical population genetics model assuming a panmictic, single-sex population of infinite size (for full details, see *SI Appendix*, *Supplementary Methods* and ref. 24). The model considers a single autosomal locus with three alleles: the wild type, a genetic construct containing a genomic editor capable of editing the wild-type allele, and the edit created by the editor. The frequency of the construct in the population is denoted by q, 0≤q≤1. For simplicity, we assume the edit causes embryonic lethality, and frequencies are measured after this has occurred (e.g., at the hatchling stage), when all edited individuals have died and there are only 2 alleles (wild-types and editor). If the editor has no fitness effect when heterozygous and causes complete lethality or sterility when homozygous, then the selection coefficient against it due to this effect is equal to its frequency in the population (*q*), and its fitness due to this effect is $w_{\mathrm{Rec}}=1-q$ (Fig. 1*A*, orange line). If the editor also acts in the germline of heterozygotes to convert the wild-type allele into a dominant embryonic lethal, then that will increase its frequency and its fitness due to this effect is $w_{\mathrm{Dom}}=\frac{1}{1-q}$ (red line), precisely balancing the fitness effect due to recessive lethality, giving an overall fitness of $w_{\mathrm{Total}}=w_{\mathrm{Rec}}*w_{\mathrm{Dom}}=1$ across the full range of frequencies (blue line; full details of the derivation given in *SI Appendix*, *Supplementary Methods*). Despite the editor being selectively neutral, it still imposes a reproductive load (L) on the population that increases with frequency, where $L=\frac{2q}{1+q}$ (Fig. 1*B*, blue line). This load arises due to both the recessive lethality of the construct, acting to remove homozygotes (Fig. 1*B*, orange line), and the action of the protected editor generating unprotected dominant lethal edits in heterozygotes (Fig. 1*B*, red line).

In principle, a genomic editor with the above features (causing death or sterility of individuals carrying two copies of the construct and of the progeny of heterozygous carriers not inheriting the construct) could be built in multiple ways. Following previous proposals (23, 25), one could create a genetic construct that 1) is inserted into a haplosufficient (HS) gene required for survival in both sexes such that its presence disrupts function and creates recessive lethality or sterility, 2) encodes Cas9 and gRNA that act exclusively in the germline to create dominant knockout mutations in a haploinsufficient (HI) gene that is required for survival or reproduction but is not needed in the germline after expression of the editor, and 3) contains a functional recoded version of the target gene resistant to editing that rescues the dominant fitness effects of a single copy of the edit (Fig. 1 *C*, *Upper*). A simpler approach would be to insert the construct into a HS gene required for survival or fertility in both sexes which can also be edited by the genomic editor to create a dominant-negative or gain of function mutation (26–30). These kinds of mutations create a protein which either interferes with the normal function of the wild-type protein or takes on a different function, which if expressed in heterozygotes can cause dominant lethality or sterility. In this design, if the construct is inserted such that its presence creates a premature stop codon upstream of the dominant-negative edit, the construct will prevent expression of the edited allele located on the same chromosome, and thus provide protection against its dominant-negative effects (Fig. 1 *C*, *Lower*). This arrangement would obviate the need for the construct to contain a recoded version of the target gene, potentially making it significantly simpler to engineer. In this paper, we explore the performance and robustness of this single-locus design, referred to as a protected dominant-negative editor (PDNE), and further consider alternative two-locus designs in *SI Appendix*, *Supplementary Results*.

### 1.2. Performance.

We next assess the performance of the PDNE by simulating releases of males heterozygous for the construct into a single, well-mixed population with two life stages, juveniles and adults, with density-dependent mortality occurring at the juvenile stage. For comparison, a range of alternative self-limiting genetic strategies currently used or in development are also modeled, including the release of

a. Males homozygous for dominant lethal genes in which
  I. the gene affects survival of both sexes before density-dependent mortality, equivalent to the sterile male technique (SIT; 31, 32) or individuals carrying *Wolbachia* leading to incompatibility (33, 34).
  II. the gene affects survival of both sexes acting after density-dependent mortality, equivalent to a transgenic knockout of a gene required, for example, for pupal to adult maturation (RIDL; 35–38).
  III. the gene causes lethality in females only and acts after density-dependent mortality (fsRIDL; 39–41).
b. Males homozygous for an autosomal X-shredder (XS) which causes sex-ratio distortion toward males (42–44); homozygous males could be created if, for example, the X-shredder is repressible in the lab.
c. Males carrying a Y-linked editor (YLE) which creates dominant lethal female-specific edits, or an autosomal dominant lethal allele that causes lethality in females and drives via homing in males (fs-RIDL-drive; 14, 27, 45).

**Fig. 2.**
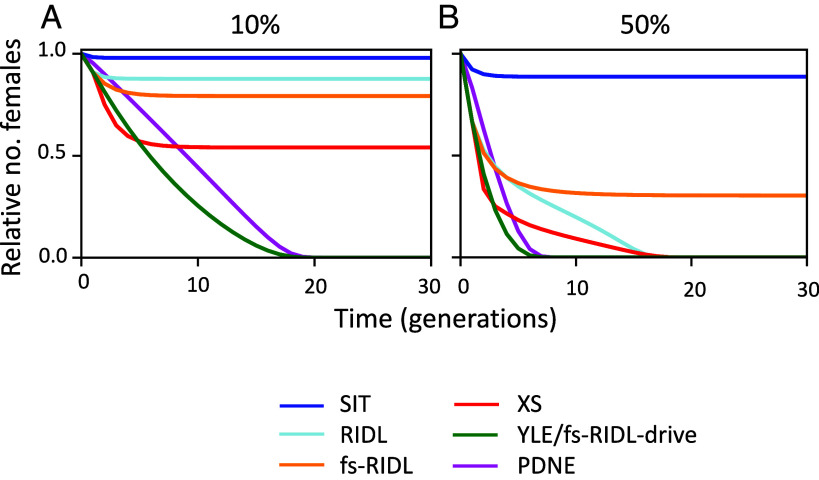
Time-course simulations of the relative number of females following repeated releases each generation of males carrying different constructs at (*A*) 10% and (*B*) 50% of the original male population size. Strategies include release of sterile males that result in death of offspring before density-dependent mortality (SIT, blue), males homozygous for either a dominant lethal allele which causes death after density-dependent mortality in both sexes (RIDL, light blue) or only females (fs-RIDL, orange), a sex-ratio distorter (XS, red), or males carrying one copy of the Y-linked editor (green) or PDNE (pink), assuming all costs cause death after density-dependent mortality. All strategies are modeled with idealized parameters in a population with an intrinsic rate of increase of *R_m_* = 6. The PDNE was simulated using a single locus design; however, in idealized conditions, the results are equivalent for two-locus implementations.

**Fig. 3.**
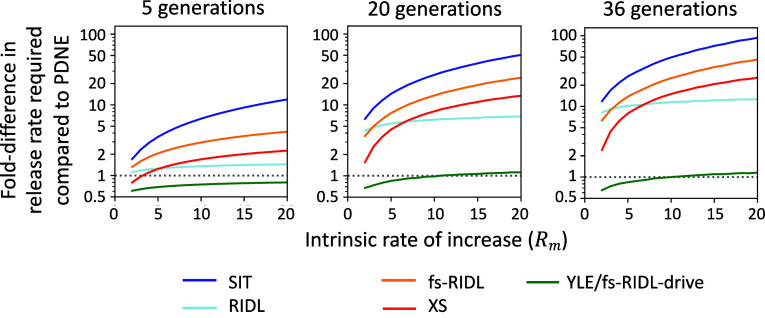
The fold difference in the number of males required for release each generation to suppress the number of females in a population with a range of intrinsic growth rates by 95% for a range of alternative strategies (described in Fig. 2) compared to releasing PDNE males. Parity to the PDNE is indicated by the gray dotted line. From left to right, plots show varying time frames in which the 95% suppression is required. Fold differences are calculated by dividing release rates for each strategy by the release rate required for the PDNE to achieve equivalent levels of suppression. All strategies are modeled with idealized parameters.

For each of these strategies, we modeled the idealized case of perfect genetic efficiencies and no unintended fitness effects. Fig. 2 shows the relative numbers of females in a population following repeated releases of modified males each generation at 10 or 50% of the original male population size. The combination of persistence and load of the PDNE means that it is substantially more efficient than SIT, RIDL, fsRIDL, and XS, where at both release frequencies it can eliminate the population while the alternatives either cannot or require more generations to do so. Rather, the suppression achieved by the PDNE is comparable to the previous best in class YLE or fs-RIDL-drive. This improvement can be quantified by comparing the numbers of males needed to be released each generation to achieve a certain level of suppression. This metric can be used to evaluate efficiencies for populations or species with different capacities to reproduce at low density (their intrinsic rate of increase, *R_m_*) and for a variety of different suppression goals. For example, releasing PDNE-carrying males each generation at 5.2% of the original male population size is sufficient to achieve 95% suppression within 36 generations in a population with an *R_m_* of 20, offering a 94-fold increase in efficiency compared to SIT. Improved efficiency is observed in populations with a range of different values of *R_m_* and when varying the level of suppression of the female population or timeframe in which the suppression is required (Fig. 3, Table 1, and *SI Appendix*, Fig. S1). The greatest reductions in release rate requirements compared to alternative strategies occur when there is a longer timeframe for the suppression goal to be achieved and in populations with higher *R_m_*. If the level of suppression required is lower, even greater gains in efficiency are observed (Table 1 and *SI Appendix*, Fig. S1). Finally, although the most efficient target gene would be one which affects survival after density-dependent mortality occurs, genes which affect survival early in development or impact fertility can also offer effective suppression (*SI Appendix*, Fig. S2).

**Table 1.** The number of released PDNE heterozygous males per generation (as a proportion of the starting male population) required to suppress the level of females by 67, 95, or 99% within 5 or 36 generations in a population with an intrinsic growth rate of 2, 6, or 20

	Intrinsic rate of increase (*R_m_*)		
	2	6	20
Level of suppression within 36 gens			
67%	0.017 [16]	0.03 [43]	0.036 [135]
95%	0.023 [12]	0.042 [31]	0.052 [94]
99%	0.025 [11]	0.044 [30]	0.056 [87]
Level of suppression within 5 gens			
67%	0.217 [2]	0.303 [6]	0.344 [20]
95%	0.481[2]	0.685 [4]	0.819 [12]
99%	0.693 [1]	0.992 [3]	1.228 [9]

Square brackets show the fold reduction in release rates compared to the sterile insect technique. All model parameters are idealized.

### 1.3. Robustness of Performance and Behavior.

**Fig. 4.**
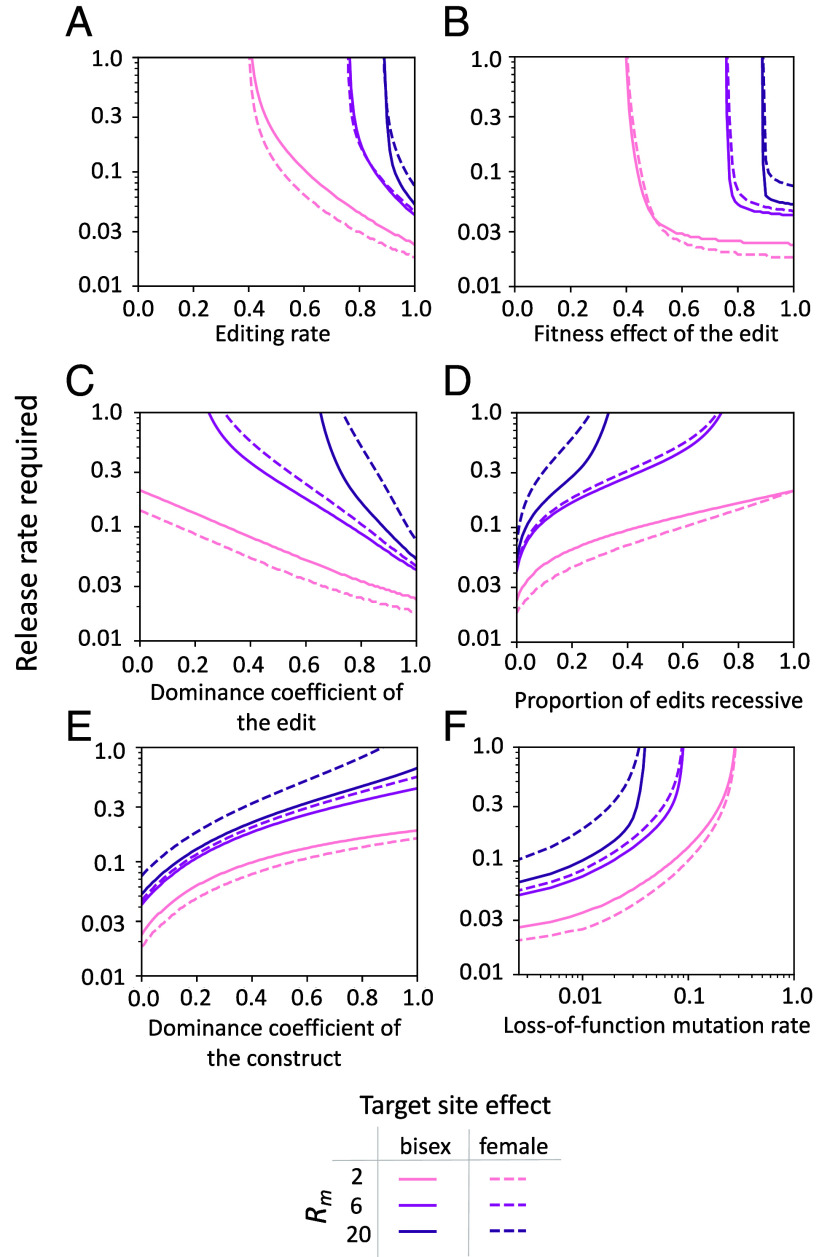
The number of released heterozygous PDNE males per generation (as a proportion of the starting male population) required to suppress the number of females in a population by 95% within 36 generations when varying parameters associated with the construct activity, fitness costs, and stability, including (*A*) the probability the construct creates an edit, where all edits created are dominant, (*B*) the fitness cost of the edit, (*C*) the dominance coefficient of the edit, (*D*) the fraction of edits the editor creates which are recessive rather than dominant, (*E*) the dominance coefficient of the construct itself, and (*F*) the probability each construct component acquires a loss-of-function mutation each generation. Release rates are shown for populations with intrinsic rates of increase of 2 (peach), 6 (magenta), and 20 (purple) and for designs in which the dominant edit created by the PDNE affects both sexes (solid lines) or only females (dashed lines). All other parameters are idealized.

In practice, it will not be possible to engineer a construct which behaves in the idealized way we have modeled, and therefore, it is important to explore the impact of possible imperfections on its performance and behavior. The two most important impacts to consider are, first, how various deviations may affect the efficiency of the system—the release rates required—and, second, whether there are any molecular inefficiencies that could cause the construct to drive and no longer remain localized. Considering first the effects on efficiency, Fig. 4 *A*–*D* shows the release rates required to suppress a population with an *R_m_* of 2, 6, or 20 by 95% within 36 generations while varying the editing parameters, keeping all other parameter values as ideal. When parameters associated with creation of the dominant edit are suboptimal, such as the editing rate and fitness costs of the edit, the balance is tipped in favor of selection, increasing the release rates required to achieve the desired level of suppression. Interestingly, similar suppression efficiencies can be achieved if the edits created only affect female fitness, and in some cases, this can even provide a gain in efficiency (Fig. 4, dashed lines). Assuming the cost of the edit is fully dominant, the release rates required are little affected by some residual fitness in individuals carrying the edit (e.g., up to 10% fitness for *R*_*m*_ = 20, and 50% fitness with *R_m_* = 2; Fig. 4*B*). Above these thresholds the strategy fails. By contrast, release rates required respond more gradually with deviations from complete dominance (Fig. 4*C*). If, rather than all edits having the same fitness effect, a proportion cause complete recessive lethality or sterility, this increases release rates more severely than an equivalent deviation in dominance that affects all edits (Fig. 4*D*). In *Drosophila,* most recessive lethal alleles have at least some small heterozygous fitness effects (46) and in previously engineered gene drives leaky expression of Cas9 causing editing in the soma has also led to unintended fitness costs in heterozygotes (47, 48). If there is increased lethality in individuals heterozygous for the PDNE, due to costs associated with disruption of the construct insertion site, expression of the editor, or off-target editing, then selection against the construct is increased, and consequently, release rate requirements are greater (Fig. 4*E*).

To assess the possibility that some imperfection in the construct might lead to spread through a population and control no longer being localized, we first return to our analytical model to explore the conditions under which the relative fitness of the construct remains below one at all construct frequencies, and thus drive is prevented. In the case where all edits produced by the editor are fully dominant and penetrant lethals (i.e., the edit responsible for the driving force has maximal effect), the construct is not expected to drive as long as $\frac{s-hs}{1-hs}\geq u$, where s is the fitness cost in homozygotes, hs is the fitness cost in heterozygotes, and u is the editing rate. It follows that if the recessive costs of the construct are also fully penetrant lethals (s=1), the condition is satisfied and drive is prevented, though other parameter combinations would also suffice. Second, once released it is also possible for the components of the construct to acquire loss-of-function mutations that change the construct’s behavior. To check whether any deletion-derivative constructs can spread, we simulated releases of the construct with 1% chance of each molecular component (Cas9 and gRNA) losing function each generation. Our results show that none of the alleles containing nonfunctional components can drive from low frequency and all remain below the release frequency of the original PDNE after single or repeat releases (*SI Appendix*, Fig. S3). Rather, loss-of-function mutations reduce the efficiency of the strategy since genomic editing is not possible without both the Cas9 and gRNA, leading to fewer dominant edits being created, reducing both persistence and load (Fig. 4*F*).

### 1.4. Boosting Efficiency.

**Fig. 5.**
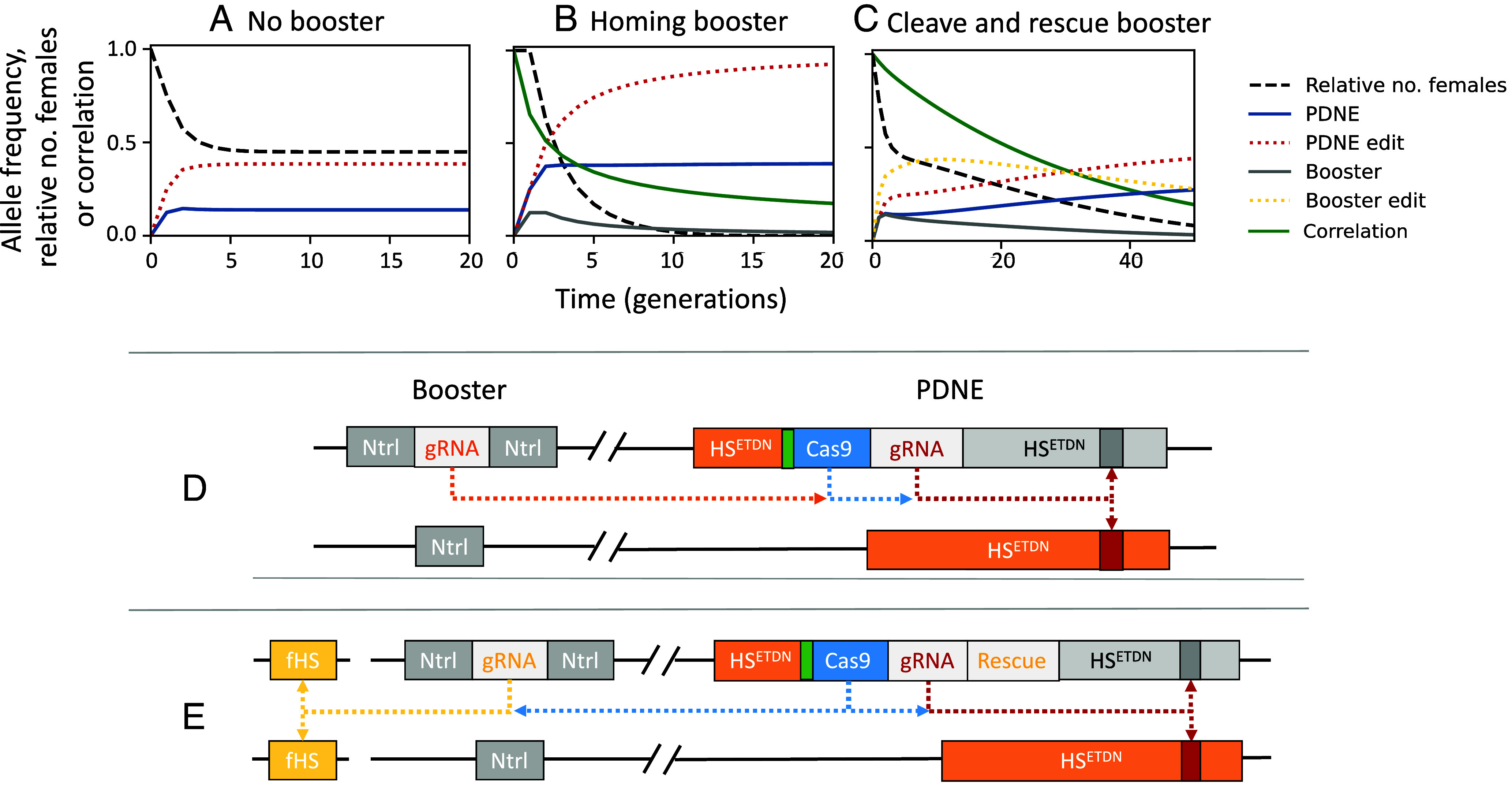
Time series simulations of single releases of males heterozygous for an idealized PDNE with or without homing or cleave and rescue booster constructs at 100% of the initial male population (*A*–*C*) and possible CRISPR-based molecular configurations (*D* and *E*). When the PDNE is released in males that also carry an unlinked homing-based booster that allows the PDNE to home in its presence (*B*), the PDNE (blue, solid) reaches a greater frequency than it would do without the booster (*A*). Since the booster (gray, solid) is inherited in a Mendelian manner, it is gradually lost over time, and therefore, boosting remains temporary. The higher frequency reached by the PDNE, and the higher frequency of edits (red, dotted) causes a greater load on the population and consequently causes a greater level of suppression (black, dashed). Panel (*C*) shows an alternative approach where a female-specific PDNE (blue, solid) is released in males which also carry a cleave and rescue booster (gray, solid) that creates edits in an unlinked gene to disrupt its function (yellow, dotted). In this example, the female-specific HS edits created by the booster act to reduce the fitness of individuals (or their descendants) who would have otherwise survived the PDNE-created female-specific HI mutation (i.e., males). If the PDNE is designed such that it rescues the function of these edits the presence of the booster will increase the selective advantage of the PDNE over the wild-type, increasing its frequency. Consequently, the PDNE reaches greater frequencies and inflicts a greater load on the population, increasing the level of suppression achieved. In this example, the two constructs are linked (r = 0.05). High correlation between the PDNE and booster due to linkage upon release (green, solid) facilitates persistence of the booster, increasing boosting. Over time recombination breaks down the correlation, and the booster decreases in frequency when inherited in individuals who do not carry the protective PDNE and who succumb to the fitness effects of either of the costly edits. Correlation is calculated as $\frac{\left(p_{GB}-p_{G}*p_{B}\right)}{\sqrt{p_{G}\left(1-p_{G}\right)p_{B}\left(1-p_{B}\right)}}$, where $p_{\mathrm{GB}}$ is the frequency of chromosomes containing both the genomic editor and booster, and $p_{G}$ and $p_{B}$ are the frequency of the editor and booster respectively. Diagrams (*D*) and (*E*) show possible molecular configurations of the two boosting strategies. A homing-based booster can be engineered using a gRNA targeting the insertion site of the PDNE and can be linked or unlinked to the PDNE construct. A cleave and rescue booster can similarly be constructed by 1) engineering the booster to contain a gRNA which guides the Cas9 expressed from the PDNE to create a costly edit at a second functional target gene and 2) modifying the PDNE to contain a cleavage-resistant recoded copy of the target gene that rescues function. To facilitate comparison, all simulations involve a PDNE that induces female-specific dominant edits, although the homing-based booster can also effectively boost a bisex design too.

Though our design offers significant improvement in efficiency over alternative strategies, further reductions in release rate requirements can be made by releasing the construct alongside one or more “booster” constructs that facilitate a temporary increase in the frequency of the effector construct (49, 50). One approach is to release a booster which allows the effector construct to home in its presence but itself is inherited in a Mendelian manner and is therefore lost over time rendering boosting temporary. A homing-based booster could be implemented by simply releasing a second construct containing a gRNA that combines with the Cas9 expressed from the PDNE to cleave the WT version of the PDNE insertion site, relying on the cell’s homology-directed repair machinery to home the PDNE (Fig. 5*D*). Fig. 5*B* shows a time series simulation of a single release of individuals heterozygous for the PDNE and one copy of the homing-based booster at an unlinked locus. The PDNE (blue) increases in frequency in the presence of the booster (gray) which gradually disappears. With repeated releases, a homing-based booster can reduce the numbers of males needed to be released each generation by up to 10-fold (*SI Appendix*, Figs. S4 and S5). For instance, releasing the PDNE alongside two copies of a booster reduces the required release rate to achieve 95% suppression of a population with an *R_m_* = 6 within 36 generations from 4.4 to 0.4%. Compared to an optimal version of SIT, this approach can be three orders of magnitude more efficient (*SI Appendix*, Fig. S4). Greater persistence of the PDNE means that boosting it, rather than alternative load-inducing constructs, can be considerably more impactful, offering up to a 100-fold increase in efficiency compared to boosting an X-shredder or fsRIDL construct (*SI Appendix*, Fig. S5). In species where homing rates are low, an alternative approach could involve a cleave and rescue booster which increases the frequency of the effector by killing individuals which do not inherit it and therefore providing a fitness advantage (Fig. 5 *C* and *E*).

## 2. Discussion

Given that the inefficiency of SIT is due to the disappearance of the causative agent within a single generation, an obvious way to improve efficiency is to increase its persistence. Here, we have proposed a way of increasing persistence of a costly allele by counterbalancing negative selection with genetic drive such that the construct is selectively neutral despite imposing a reproductive load. In our design, although drive occurs at the level of individual inheritance, it does not translate into population spread. Once released, the combination of load and selective neutrality allows our construct to act, at least in the idealized case, like a genetic perpetual motion machine, suppressing the population indefinitely without the need for additional releases. In reality of course, achieving perfect balance (like perpetual motion) will not be possible—editing rates will not be 100%; heterozygous fitness effects of the insert will not be 0; mutation rates of the construct will not be 0—but even with some imperfections, our modeling shows that the design is expected to result in self-limiting population suppression with considerably lower release requirements than the currently utilized SIT and a range of alternative candidate strategies (see also ref. 51). Indeed, it is these very imperfections that would prevent the construct from gradually diffusing neutrally over a continuous landscape.

Our proposed design relies on three key features involving an autosomal construct that 1) causes recessive lethality or sterility in both sexes, 2) encodes a genome editor creating female-specific or bisex dominant lethal or sterile edits, and 3) provides some protection against the action or consequences of the editor. Although in principle, the design could be implemented in many ways, it is possible to engineer this genetic strategy using current molecular tools, and we have proposed simple one- and two-locus CRISPR-based cleave and rescue approaches. Other strategies with a similar molecular configuration but which differ in their dynamics include cleave and rescue (ClvR) or toxin–antidote recessive embryo (TARE) designs for population modification (22, 23) and strategies for suppression that exhibit drive (TADE; 25), the latter of which differs from our design only in that it caused recessive lethality or sterility in a single sex rather than in both, and therefore, in the absence of other fitness costs, would increase in frequency in a population. Other strategies with nondriving, selectively neutral dynamics similar to our design but which take a different molecular approach include YLEs and fs-RIDL-drives (14). YLEs are genomic editors which generate costly edits that affect females and achieve selective neutrality due to being located on the Y chromosome where they are hidden from selection occuring in females. In contrast, the fs-RIDL-drive construct is autosomal and causes dominant costs in females that inherit it. Selective neutrality is achieved similarly to the PDNE, by balancing selection against the dominant lethality in females with homing-based genetic drive in males. Although both strategies offer comparable efficiency to the PDNE, the YLE requires integration into, and germline expression from, the Y-chromosome, which can be difficult to achieve (52–54) and also restricts the approach to species with separate sexes. The efficiency of the fs-RIDL-drive strategy depends on high homing rates which in some species are difficult to achieve (55–57). Our proposed design evades these difficulties since it does not require homing, can be built using autosomal loci in species with or without sex chromosomes, and does not require sex-specific fitness effects and therefore may be easier to engineer.

Our single-locus PDNE configuration (Fig. 1 *C*, *Lower*) offers a particularly attractive option for construction since it prevents the need to include a recoded copy of a gene within the construct, which in some cases has been difficult to achieve (58). The design relies on integrating the construct into a haplosufficient gene needed in both sexes in which it is possible for the genomic editor to create a dominant-negative mutation. A search of FlyBase indicates there are 45 genes in *Drosophila melanogaster* with both dominant lethal and recessive lethal mutations, providing a starting point to identify a suitable gene. *Doublesex* is a noteworthy gene involved in insect sex determination for which both bisex recessive and female-specific dominant sterile mutations have been generated (27, 28), and also forms the basis of a range of emerging synthetic genetic control strategies for suppression (8, 16, 45, 59, 60). Although in our design, we propose inserting the construct upstream of the target site to prevent expression of the dominant-negative or gain of function edit by introducing a premature stop codon, as long as the haplotype which contains the editor also contains a disrupted version of the haplosufficient gene, the construct could alternatively be located near to, but downstream of the edit, inside or outside of the target gene, or within a nearby haplosufficient gene (*SI Appendix*, Fig. S6). In cases where the disrupted version of the haplosufficient gene does not prevent expression of the edited transcript, the editor target site (or even the whole gene) located in the same haplotype could simply be deleted (*SI Appendix*, Fig. S6, *Right*), preventing the edit from being created and thus providing an alternative means of protection without incorporating a recoded gene into the construct. Note that a similar approach to alleviating the need for encoding a rescue component could be taken to engineer alternative cleave and rescue designs with other objectives. For example, a TADE suppression design could be constructed using a single sex-specific haplosufficient gene in which dominant-negative or gain of function edits can be made (25).

In our alternative two-locus molecular configurations, the construct is inserted into a haplosufficient gene and the editor engineered to target a separate gene elsewhere in the genome (Fig. 1 *C*, *Upper*). This approach would allow for the editor to create a dominant edit by knocking out a haploinsufficient gene, therefore broadening the potential choice of target sites. 818 genes in FlyBase are annotated as having an amorphic or loss of function allele giving a recessive lethal phenotype, suggesting ample choice for insertion sites. Cook et al. (61), (table 2) list 43 genes in *D. melanogaster* that are haplolethal or haplosterile, plus another six regions of the genome where haplolethal or haplosterile mutations can occur but the exact gene had not yet been identified, offering a range of putative target genes. If the two genes are closely linked, protecting the construct against the edit could be achieved by recoding the target gene in situ, whereas if they are unlinked, it would need to form part of the construct.

Regardless of the construct’s precise configuration, as with all forms of pest control attention needs to be given to the potential for resistance to arise. Since, at least in our designs, the strategy relies on a genomic editor, there is potential for the editor to make mistakes and generate unwanted edits (62, 63). If some have fitness effects that differ from the desired dominant edit and are resistant to future cleavage they can reduce the strength of drive and lower the efficiency of the construct. In the case that some edits have no fitness costs, they are expected to be subject to strong positive selection likely to result in the failure of the strategy. If instead, some edits have recessive fitness effects, our sensitivity analysis shows that they can significantly reduce efficiency, though do not totally prevent suppression, at least in the absence of the resistant allele homing. Thus, efforts should be made to prevent the production of unintended edits. Following from previous suggestions, the probability of creating unwanted edits could be mitigated by targeting a highly conserved gene or sequence, or by using multiple gRNAs or different Cas enzymes (16, 19, 23, 28, 64–66). Creation of C-terminal frameshifts with dominant effects would likely be the most robust approach in terms of allowing multiple gRNAs and retarding resistance. One class of target genes where this is more likely to occur is those where the proteins act as multimers (i.e., by exploiting the phenomenon of multimer poisoning) (29, 30), with *doublesex* being a likely example. In principle, a certain level of preexisting polymorphisms in the target population could also be overcome by using multiple gRNAs to the different preexisting sequences. One could also increase the likelihood of making a precise sequence change by using prime or base editing (67–69) or by releasing the desired sequence alongside the construct for use as homology-directed repair template (27). Finally, in addition to preventing the generation of unintended edits, restricting editing to the germline by avoiding somatic expression of the nuclease will help maximize efficiency by avoiding unwanted fitness costs in construct heterozygotes.

To guarantee that the construct and its impacts remain localized, it would be good to ensure that the recessive costs of the construct are both evolutionarily stable and 100% penetrant. Our proposed designs induce recessive lethality or sterility by inserting the construct into or linking it to a disrupted allele of a HS gene required for survival or fertility (Fig. 1 and *SI Appendix*, Fig. S6). With these designs, loss-of-function mutations in any of the construct components will not affect the strength of selection, and therefore, none of the construct derivatives are expected to drive. An alternative molecular design could involve inserting the construct into a neutral site and including a second gRNA which creates recessive edits at a separate HS gene, but loss-of-function mutations in this gRNA would decrease selection and therefore create deletion derivatives more likely to spread. Furthermore, to prevent drive it will be important to ensure that the editor does not gain a transmission advantage through homing when the edit is created, whether by ensuring sufficient distance between the editor and target site or by using prime- or base-editing. Additionally, an enhanced transmission advantage for the construct could occur if the killing of offspring which carry an unprotected edit results in reduced sib competition (through for example acting early in development), and therefore, efforts should be made to prevent this. Some studies have found that parental deposition of the Cas9 and gRNA can either hinder or enhance drive (22, 23, 70). In our designs, we expect deposition to increase selection, rather than drive, since cleavage of the WT target allele in construct-bearing offspring would increase selection against the construct, whereas cleavage in non-construct-bearing offspring would be redundant since they already inherit an intended dominant lethal mutation. Finally, wild populations may deviate from the random mating assumption made so far, instead exhibiting some degree of inbreeding. In these cases, we would not expect drive to occur since selection against the recessive costs of construct would be expected to increase (due to homozygotes for the construct occurring more often than in Hardy–Weinberg frequencies), and therefore, the construct is more likely to decline in frequency.

Previous studies have demonstrated that for strategies involving two constructs at separate loci, the degree to which the two loci are linked can influence their dynamics, and for this reason, genetic distance can be tuned to control persistence and efficiency (71, 72). This feature also applies to our designs involving releasing the PDNE alongside a homing or cleave and rescue booster, though the quantitative details between designs differ. Interestingly, we find that a homing booster can be located within the same gene as the PDNE, essentially forming part of the same construct, and boosting will remain temporary as long as the booster does not cause itself to home (*SI Appendix*, Fig. S7). This design may be particularly attractive when performing a risk assessment, since only a single genetic construct would need to be evaluated, or for geographically restricted suppression gene drive trials since the construct exhibits drive temporarily. In theory, our boosted designs could also form the basis of a low-threshold geographically restricted double drive for suppression, and due to the effector construct’s selective neutrality may offer increased resilience to resistant alleles compared to alternative designs (71, 73). In principle, further control of our construct’s dynamics could be achieved by using seasonally important genes (74) or environmental application of chemicals that affect the fitness effects of the construct. If the gene used to induce recessive costs is only needed periodically or not needed in the presence of the chemical, then during specific times or in the presence of the chemical, drive will outweigh selection, and allele frequencies will temporarily increase. Alternatively, if such a gene was used to induce the dominant costs, selection would outweigh drive, and the construct would be purged from the population under certain conditions. Finally, though our design may offer some implementation advantages by avoiding the need for targeting sex-specific genes, it still requires creating edits with dominant effects which can be difficult to work with. Choosing genes with functions that can be reconstituted in the laboratory might make this easier and facilitate large-scale production by allowing for pure-breeding lines and alleviating the need for time-consuming screening. For instance, using gene disruptions that are auxotrophic and able to be supplemented through diet (75–77) would allow the recessive and/or dominant costs associated with the PDNE to be absent during rearing, allowing for heterozygous and/or homozygous males to be produced without the need for crossing multiple stock lines of specific genotypes.

In summary, our proposed approach would expand the current genetic biocontrol toolbox offering alternative cleave and rescue designs for achieving highly efficient localized suppression. While most current engineered homing and toxin–antidote constructs mimic naturally evolved drive systems, our design is not expected to have evolved naturally, but rather is a synthetic combination of available molecular building blocks motivated by and tailored to a specific end use—localized suppression. Given the success in using CRISPR/Cas9 to create previously proposed designs, it is likely that the construct(s) can be built using currently available molecular tools. Which of our proposed molecular configurations would be most applicable to different species and control goals will depend on the biology of the target population, the way in which harm is inflicted by the pest, and the genes and technologies which are available within the species. In the future, it will also be useful to further compare the resilience of each design to different types of resistance, unintended fitness costs and molecular instability, and to develop more context- and species-specific models to explore optimal rearing and release strategies and compare the potential for dispersal from release sites across designs.

## 3. Materials and Methods

To explore the dynamics of a genetic construct which creates dominant lethal edits in the genome and is protected against them, we first developed a simple single-locus, single-sex population genetics model which could be analyzed analytically. We then extended this model, developing a series of more complex models which incorporate additional features including density-dependent population dynamics, sex specificity, multiple loci, and the capacity to model a series of alternate localizable genetic biocontrol strategies for comparison. Since the extended models were too complex to usefully analyze analytically, they were assessed using simulations. A full description of the models and parameters used can be found in *SI Appendix*, *Supplementary Methods*.

## Supplementary Material

Appendix 01 (PDF)

## Data Availability

Computer code data have been deposited in https://doi.org/10.5281/zenodo.14446582 (24).
