# Supplementary material for: Engineering drive–selection balance for localized population suppression with neutral dynamics
Source: Proc Natl Acad Sci U S A. 2025 Feb 4;122(6):e2414207122. doi: 10.1073/pnas.2414207122 (PMC11831207; doi:10.1073/pnas.2414207122)
Supplement: Supplementary file 1 — Appendix 01 (PDF) [file pnas.2414207122.sapp.pdf]

SI Appendix

Supplementary Figures

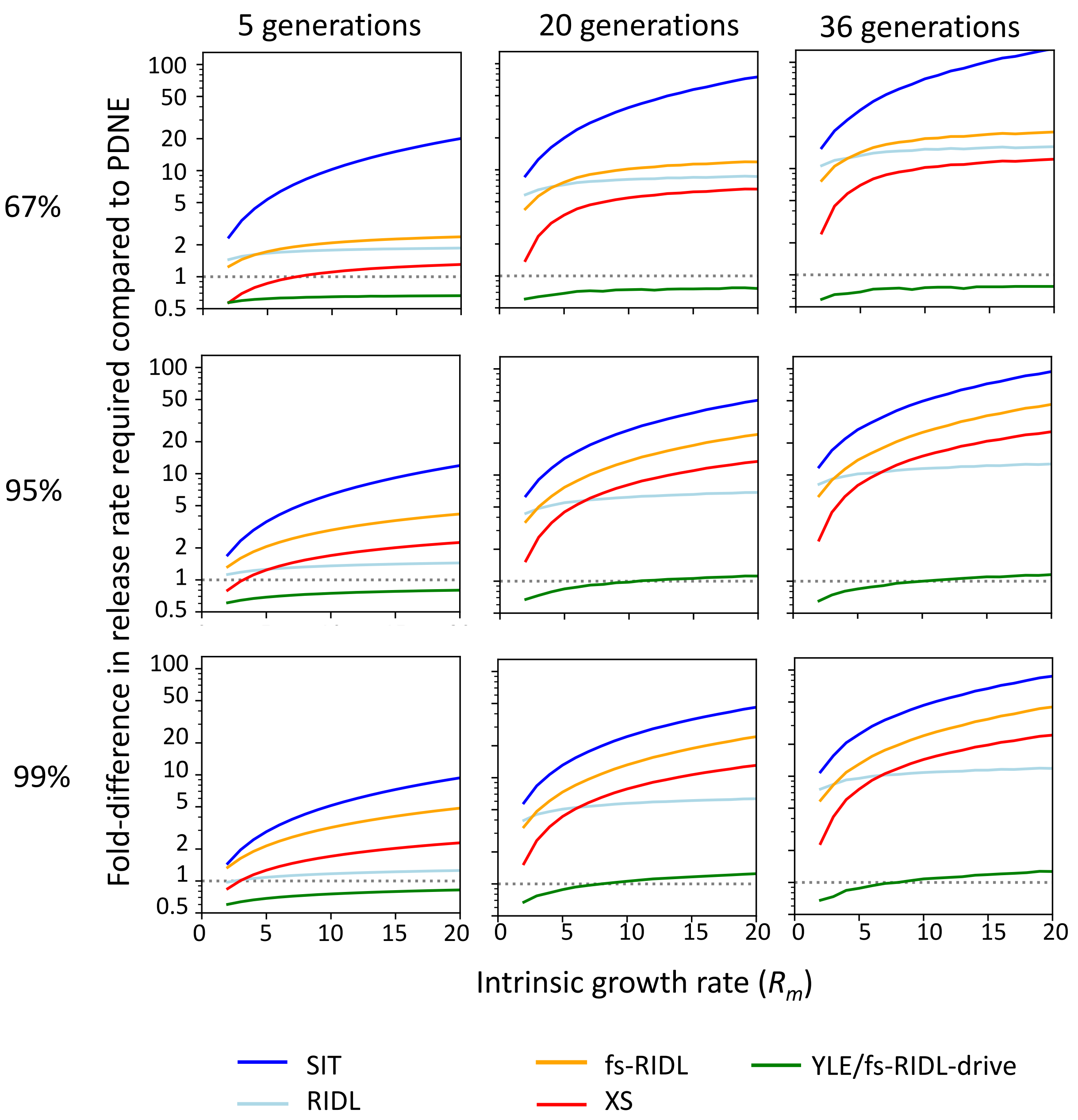

Fig. S1 – The fold-difference in male release rates required to suppress a population by 67%, 95% and 99% using a range of strategies (as in Fig. 2) compared to a PDNE as a function of the intrinsic growth rate of the target population. From left to right plots show varying time frames within which the level of suppression is achieved. Parity to the PDNE is indicated by the grey dotted line. All strategies are modelled with idealised parameters.

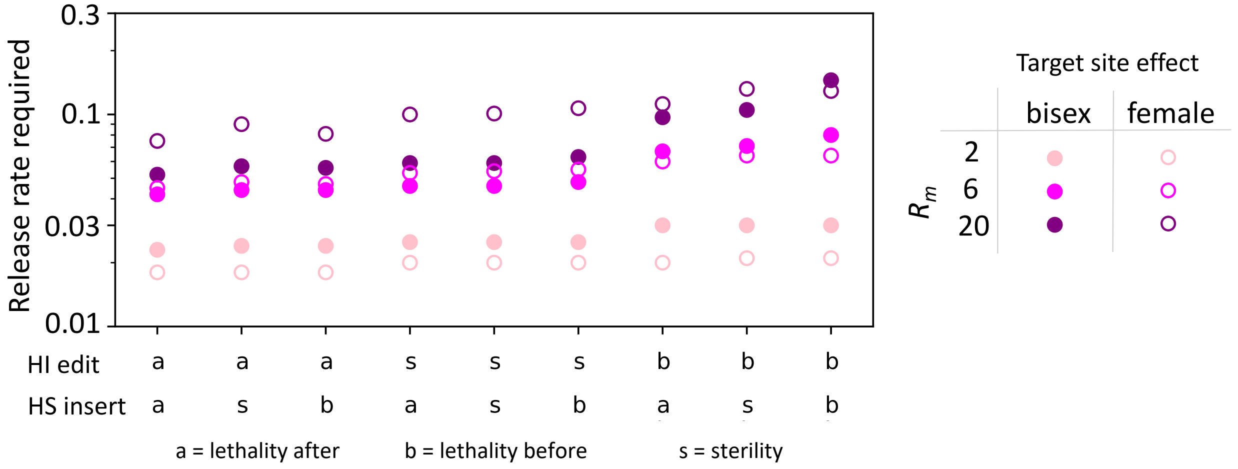

Fig. S2 – The release rates required to suppress the number of females in a population by 95% within 36 generations when varying whether the dominant edit and recessive disruption causes sterility (s) or lethality before (b) or after (a) density dependent mortality. Release rates are shown for populations with intrinsic rates of increase of 2 (peach), 6 (magenta) and 20 (purple) and for designs in which the dominant edit created by the PDNE affects both sexes (filled circles) or only females (open circles). All other parameters are idealised.

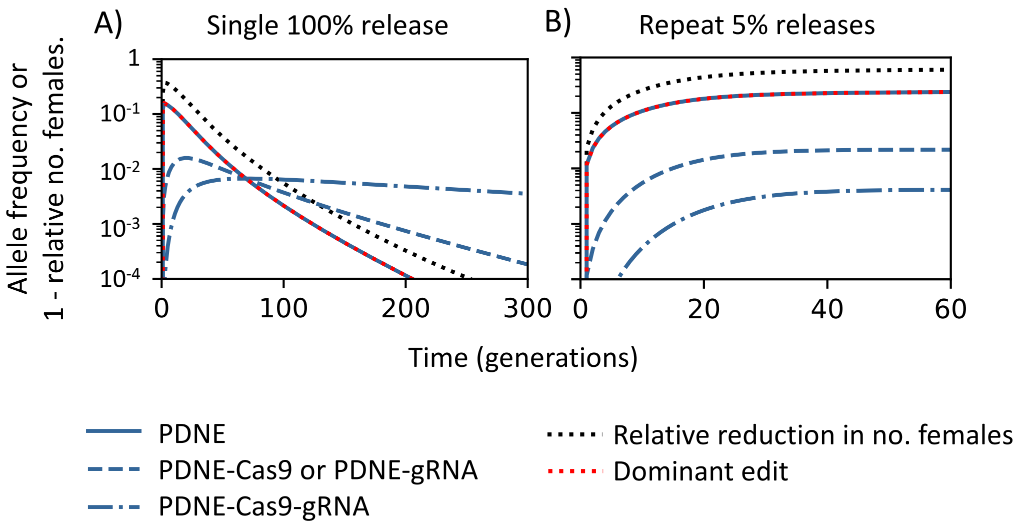

Fig. S3 – Time series simulations of **(A)** single and **(B)** repeated releases of males heterozygous for an idealized PDNE where loss-of-function mutations occur in each construct component (Cas9 and gRNA) at 1% per generation. After a single release in the first generation the intact construct (PDNE, blue solid, overlapping with the red dotted line) creates edits (dominant edit, red dotted) in the first generation after release causing an increase in the reduction of females relative to the starting population (black, dotted). In subsequent generations the intact construct decreases, owing to the construct accumulating loss-of-function mutations in either the Cas9 or gRNA (blue dashed) or both (blue dot-dashed). None of the derivative constructs generated through loss-of-function mutations are able to drive and each remain below the frequency of the released construct in both single and repeat release regimes.

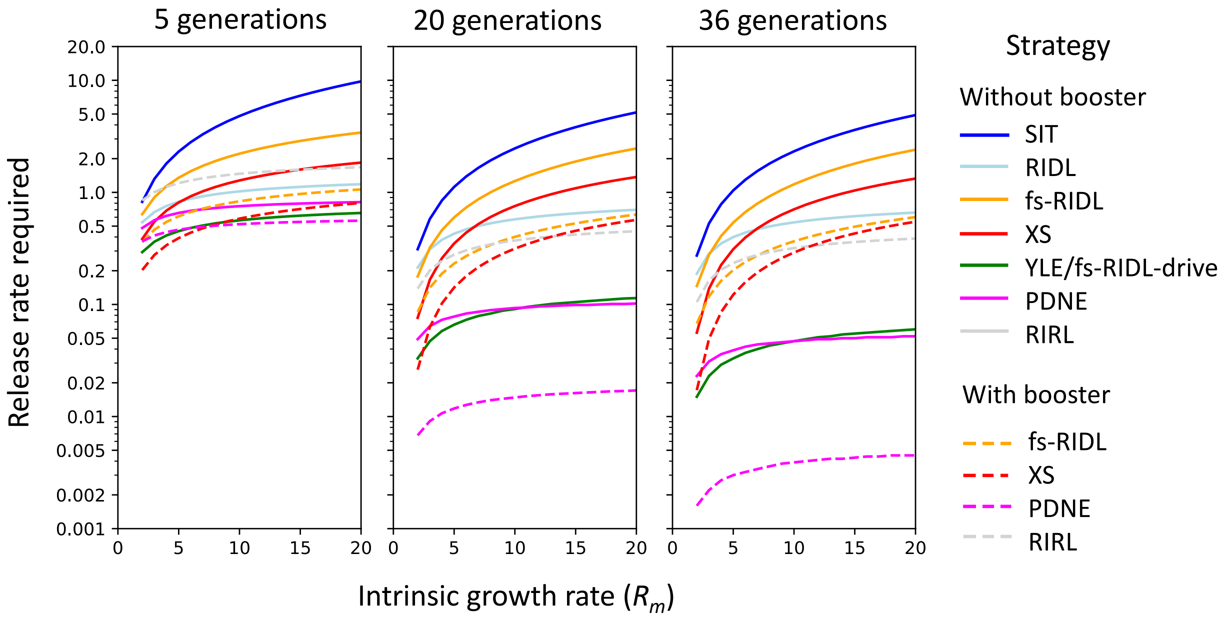

Fig. S4 – Release rate requirements to suppress a population with a range of intrinsic growth rates by 95% using a range of suppression constructs (as in Fig. 2) released without (solid) and with (dashed) a booster. From left to right plots show varying time frames in which the 95% suppression is achieved. For all scenarios we assume release of two copies of the booster unlinked to the construct. Note that since no offspring who inherit the SIT or RIDL constructs survive, the presence of a booster makes no difference to release rate requirements (not shown). Additionally, since the YLE is on the Y-chromosome and the fs-RIDL-drive homes itself, neither can be boosted via homing (not shown). Boosting in individuals carrying a recessive lethal (RIRL) was also modelled (grey), equivalent to the PDNE without the driving force, which, in the absence of the booster, is unable to suppress the population by 95% with reasonable release rates (not shown).

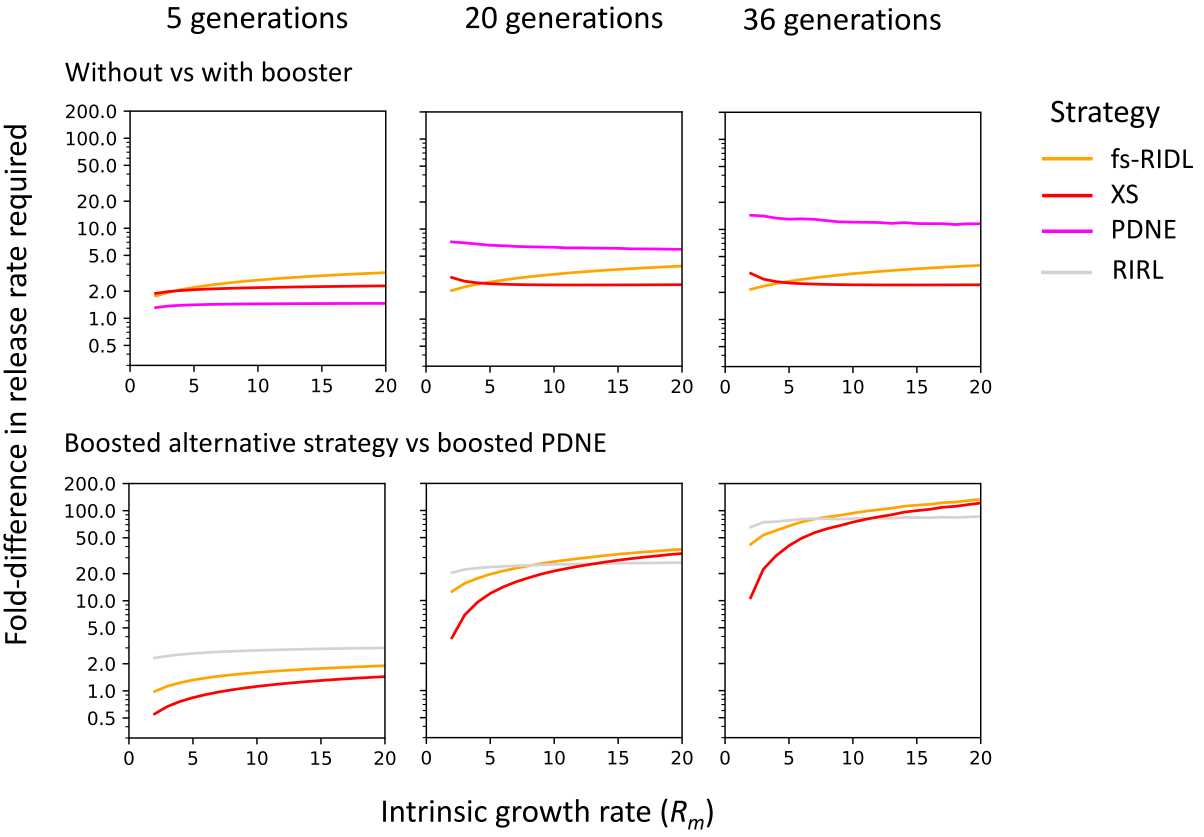

Fig. S5 – The fold-difference in male release rates required for strategies (as in SI Appendix, Fig. S4) when comparing each strategy with and without a homing booster (upper) or comparing different boosted designs to the boosted PDNE (lower). All strategies are modelled with idealised parameters. Released males are homozygous for the booster which is unlinked from the PDNE.

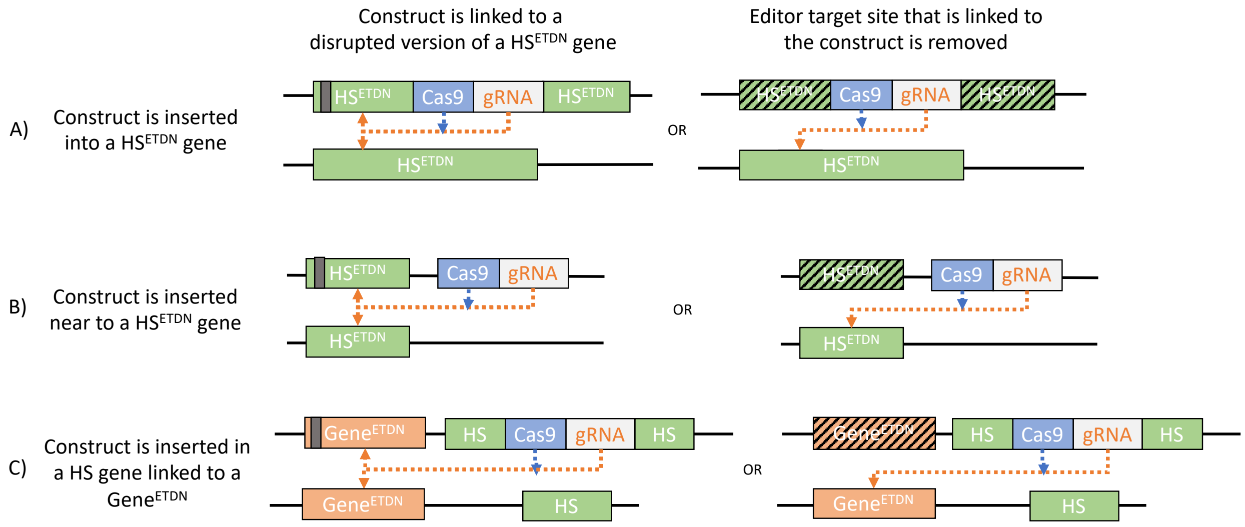

Fig. S6 – Possible CRISPR/Cas9 molecular configurations where insertion of the construct (Cas9 and gRNA) does not directly lead to recessive lethality or infertility. Rather, recessive costs could be achieved by ensuring the haplotype containing the construct also contains a disrupted version of a haplo-sufficient gene (grey box). The construct could be inserted into a haplo-sufficient gene editable to a dominant negative (HS^{ETDN}, green box) **(A)**, up- or down-stream of a HS^{ETDN} gene (**B**, down-stream shown for illustration), or **(C)** within a haplo-sufficient gene and the editor designed to target a separate linked gene editable to a dominant negative ($Gene^{ETDN}$, orange box). In cases where the insertion of the construct, or the linked disruption, does not prevent expression of the dominant negative edit from the same chromosome, the editor target site or the entire target gene on the same haplotype as the construct can simply be deleted (right column, hashed gene), preventing the edit from being made.

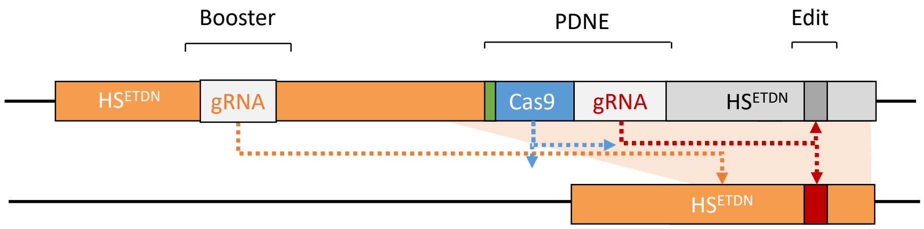

Fig. S7 – A possible CRISPR/Cas9 molecular configuration of a PDNE released alongside a gRNA which facilitates homing of the PDNE where both elements are inserted into the same HS^{ETDN} gene (orange box). With optimal parameters the dynamics of the boosted construct do not differ from the case where the booster and PDNE are unlinked (Fig. 5) as long as the gRNA responsible for boosting does not home alongside the portion of DNA containing the PDNE (with or without the dominant edit; indicated by the orange shaded area). One way of achieving this would be to ensure the booster gRNA was inserted at sufficient distance from the PDNE such that it escapes resection during homing.

Supplementary Results

Considerations for two-locus implementations

Whether our proposed design is implemented using one or two loci, the impact of suboptimal parameters associated with the edit or unintended costs in heterozygotes on the efficiency of the strategy is unchanged, at least when all other parameters are idealised. However, when constructing designs involving two loci it is important to consider the effect of linkage between the loci. For designs where the editor creates haplo-insufficient edits in a different gene to which it is inserted, and a recoded copy of that gene is located within the construct (Fig. 1C, upper, SI Appendix, Supplementary Results, Fig. S10, lower), linkage between the loci can reduce efficiency when editing rates are suboptimal (SI Appendix, Supplementary Results, Fig. S8). In contrast, in designs where the editor targets a gene closely linked to its insertion site and the recoded copy of the HI gene is in its native genomic location (SI Appendix, Supplementary Results, Fig. S10, upper), tight linkage between the construct and the HI gene is vital to maintain impact (SI Appendix, Supplementary Results, Fig. S9). For constructs that contain a recoded copy of the construct (whether in the construct or linked to it) it is possible that rescue functionality is suboptimal or that the rescue element acquires loss-of-function mutations that prevent rescue. Regardless of linkage, suboptimal rescue is expected to reduce the strength of drive since the construct will be subject to (at least some) selection against the dominant edit. This would not result in drive of the construct and instead decrease efficiency of the design, although quantifying the magnitude of this effect would require further analysis that is outside the scope of this work.

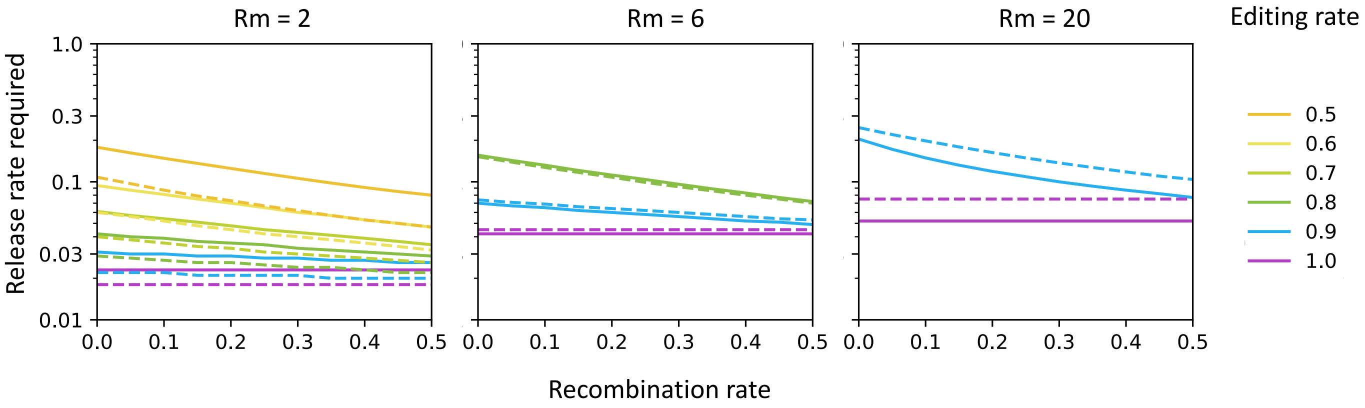

Fig. S8 – The release rates required of males carrying a PDNE implemented using two loci to suppress the level of females in a population by 95% of its original size within 36 generations as a function of the recombination rate between the PDNE and its target site and the editing rate for populations with an intrinsic growth rate 2, 6 or 20. The fitness effects of the edit created by the PDNE affect either both sexes (solid lines) or only females (dashed lines) and are assumed to cause lethality after density dependent mortality. Recall from Fig. 4 that for some editing rates suppression to the desired level is not achievable with release rates less than one, therefore not all editing rates are relevant to all R_m values used. All other parameters are assumed to be idealised.

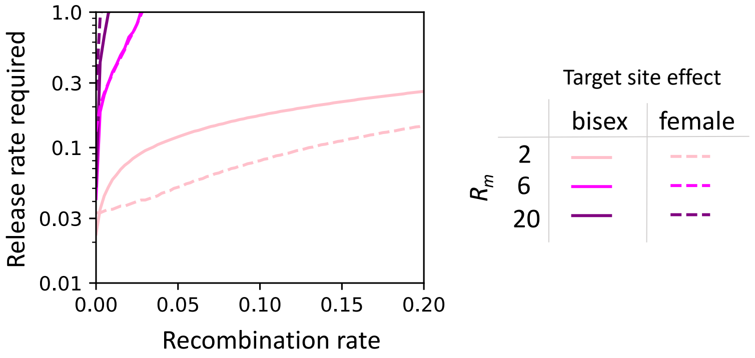

Fig. S9 – The release rates required to suppress the number of females in a population by 95% within 36 generations using a 2-locus implementation of our design where the construct causes recessive fitness costs and creates knockout mutations in a linked haplo-insufficient gene. Rescue is achieved by releasing the construct linked to a fully functional version of the haplo-insufficient gene that is resistant to cleavage.

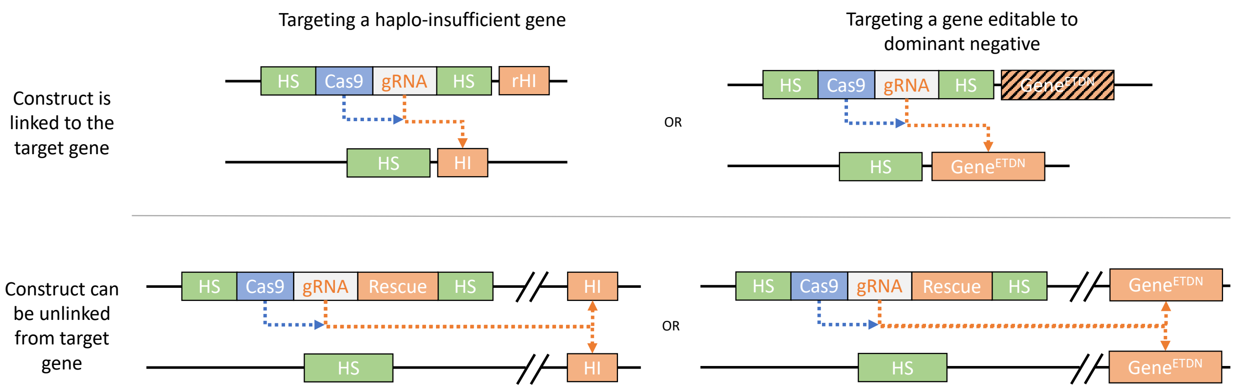

Fig. S10 – Possible CRISPR/Cas9 molecular configurations for designs using two loci. The construct (Cas9 and gRNA) can be designed to target a gene editable to a dominant negative ($Gene^{ETDN}$) or create knockout mutations in a haplo-insufficient (HI) gene, either of which can be tightly linked to the construct (top) or distantly linked (bottom), including on a different chromosome. When tightly linked, the target gene on the same chromosome as the editor can be recoded such that it cannot be targeted by the editor but is still functional (rHI). If the editor is targeting a gene editable to dominant negative the target site the whole gene linked to the construct can simply be removed (hashed). When distantly linked the construct must contain a recoded (rescue) copy of the target gene that cannot be cleaved by the editor but retains function. If the edit produces a dominant negative mutation, then the rescue may be designed to have higher expression levels, to dilute out the dominant negative protein. For illustration, the genetic constructs in the figure have been depicted as being inserted into and disrupting the haplo-sufficient gene, however, the genetic construct may also be configured to be linked to disrupted version of the HS gene (SI Appendix, Fig. S6).

Supplementary Methods

The following section describes the analytical population genetics model implemented in Mathematica and the population genetic and dynamic simulations performed using The Julia Programming language. All code required to reproduce this work has been deposited in Zenodo (https://doi.org/10.5281/zenodo.14446582).

1. One locus, single sex analytical model

1.1. The model

We first developed an analytical model to explore the dynamics of a genetic construct with recessive lethality that creates dominant lethal edits in the genome and is protected against them. The model is based on the single locus design, in which the insertion site for the construct and the target site are tightly linked within the same gene, and the construct is protected against the edits it creates on the same chromosome. The model includes a single autosomal locus with three alleles, the wild-type (W), the genomic editor (G) that is able to edit the W allele, and the edit it creates (R), and therefore 6 genotypes (WW, RR, GG, WG, WR, GR). In heterozygotes for the genomic editor and wild-type allele (WG) gamete transmission is altered by the activity of the editor, where in the germline the genomic editor converts the wild-type allele (W) to an edited allele (R) with probability u.

For simplicity and to study the fitness effects of the recessive and dominant costs independently, we consider the case where the dominant lethality of the edit acts on the embryo, preventing hatching, and the recessive lethality of the construct acts on the pupae, preventing maturation to adulthood. If we assume that the edit is fully penetrant (i.e., that all RR, WR, and GR individuals die at the embryo stage) and that mating is random then at the hatchling stage (i.e. after dominant costs and before recessive costs) the remaining 3 genotypes will be in Hardy Weinberg proportions:

$$H_{WW}(t) = p_H^2(t)$$

$$H_{WG}(t) = 2p_H(t)q_H(t)$$

$$H_{GG}(t) = q_H^2(t)$$

(1)

where $p_H(t)$ is the allele frequency of the wild-type at the hatchling stage in generation t and $q_H(t) = 1 - p_H(t)$ is the allele frequency of the genomic editor. The fraction of adults surviving the recessive fitness cost in generation t is then:

$$A(t) = p_H^2(t) + 2p_H(t)q_H(t)(1 - h_G s_G) + q_H^2(t)(1 - s_G)$$

(2)

where s_G is the fitness cost of the construct in GG homozygotes, and h_G is a dominance coefficient that describes the proportion of the cost in homozygotes that occurs in WG heterozygotes.

Thus, the load imposed on the population due to the recessive fitness costs is:

$$\mathcal{L}' = 1 - A(t)$$

(3)

and the frequencies of each genotype in the adult population are:

$$A_{WW}(t) = \frac{p_H^2(t)}{A(t)}$$

$$A_{WG}(t) = \frac{2p_H(t)q_H(t)(1 - h_G s_G)}{A(t)}$$

$$A_{GG}(t) = \frac{q_H^2(t)(1 - s_G)}{A(t)}$$

(4)

In the germline of WG heterozygotes the wild-type allele (W) is converted to an edited allele (R) with probability u. Therefore, the gamete frequencies of W, G and R alleles respectively are:

$$p_G(t) = A_{WW}(t) + \frac{A_{WG}(t)}{2}(1-u)$$

$$q_G(t) = A_{GG}(t) + \frac{A_{WG}(t)}{2}$$

$$r_G(t) = \frac{A_{WG}(t)}{2}u$$

(5)

where $r_G(t)$ is the frequency of the edited allele in gametes in generation t.

Assuming random combination of gametes during mating, the genotype frequencies in the zygotes are:

$$Z_{WW}(t+1) = p_G(t)^2$$

$$Z_{GG}(t+1) = q_G(t)^2$$

$$Z_{RR}(t+1) = r_G(t)^2$$

$$Z_{WG}(t+1) = 2q_G(t)p_G(t)$$

$$Z_{WR}(t+1) = 2p_G(t)r_G(t)$$

$$Z_{GR}(t+1) = 2q_G(t)r_G(t)$$

(6)

The fraction of zygotes without an R allele and therefore surviving the dominant costs associated with the edit and transitioning to the hatchlings stage is then:

$$H(t+1) = Z_{WW}(t+1) + Z_{GG}(t+1) + Z_{WG}(t+1)$$

Therefore, the load imposed on the population due to the dominant edits is:

$$\mathcal{L}'' = 1 - H(t+1) \tag{7}$$

and the genotype frequencies in the hatchlings are:

$$H_{WW}(t+1) = \frac{Z_{WW}(t+1)}{H(t+1)}$$

$$H_{WG}(t+1) = \frac{Z_{WG}(t+1)}{H(t+1)}$$

$$H_{GG}(t+1) = \frac{Z_{GG}(t+1)}{H(t+1)}$$

(8)

Finally, the allele frequencies in hatchings in generation $t+1$ can be calculated from the hatchling genotype frequencies:

$$p_H(t+1) = H_{WW}(t+1) + \frac{H_{WG}(t+1)}{2}$$

$$q_H(t+1) = H_{GG}(t+1) + \frac{H_{WG}(t+1)}{2}$$

(9)

Given that $p_H = 1 - q_H$ the recursion equation for the allele frequency of the genomic editor at the hatching stage is:

$$q_H(t+1) = \frac{q_H(t)((q_H(t) + h_G - q_H(t)h_G)s_G - 1)}{q_H(t)s_G(q_H(t) + 2h_G - 2q_H(t)h_G) + (q_H(t) - 1)q_H(t)(h_G s_G - 1)u - 1}$$

(10)

We can calculate the relative fitness of the genomic editor using the ratio of standardised allele frequencies across a single generation:

$$w = \frac{\left(\frac{q_H(t+1)}{1 - q_H(t+1)}\right)}{\left(\frac{q_H(t)}{1 - q_H(t)}\right)}$$

(11)

which can be expressed as a function of the allele frequency in the first generation and the model parameters:

$$w = \frac{1 - q_H(t)s_G(1-h_G) - h_G s_G}{1 - q_H(t)h_G s_G(1-u) - q_H(t)u} \tag{12}$$

The total Load imposed by the genomic editor, including both its own recessive costs and the effects of the dominant edits it creates, is:

$$\mathcal{L} = 1 - (1-\mathcal{L}')(1-\mathcal{L}'') \tag{13}$$

$$\mathcal{L} = 1 - \left(\frac{\left(q_H(t)s_G(q_H(t) + 2h_G - 2q_H(t)h_G) + q_H(t)u\big(1-q_H(t)\big)(1-h_G s_G) - 1\right)^2}{1 - q_H(t)s_G\left(2\big(1-q_H(t)\big)h_G + q_H(t)\right)} \right) \tag{14}$$

1.2. Calculating the fitness and load of the recessive and dominant effects independently

Assuming idealised parameters, when the recessive costs are present but there is no editing s_G = 1, h_G = 0 and u = 0. Substituting these values into equations 10, 12 and 14 gives the recursion equation for the allele frequency of the genetic construct in hatchlings:

$$q_H(t+1) = \frac{q_H(t)}{1+q_H(t)} \tag{15}$$

the relative fitness of the construct:

$$w_{Rec} = 1 - q_H \tag{16}$$

and the genetic load:

$$\mathcal{L}_{\text{Rec}} = q_H^2 \tag{17}$$

Alternatively, when there is genomic editing but no recessive costs, u = 1 whilst s_G = 0. Substituting these values into equations 10, 12 and 14 gives the recursion equation for the construct:

$$q_H(t+1) = \frac{q_H(t)}{1 + (q_H(t) - 1)q_H(t)}$$

(18)

the relative fitness of the construct:

$$w_{Dom} = \frac{1}{1 - q_H}$$

(19)

and the genetic load:

$$\mathcal{L}_{\mathrm{Dom}} = 1 - \left(1 + q_H(q_H - 1)\right)^2$$

(20)

Finally, we consider the case where both features are present (the construct has recessive fitness costs and also contains a functioning genomic editor), where s_G = u = 1 and $h_G = 0$. Substituting these values into equations 10, 12 and 14 gives the recursion equation for the construct:

$$q_H(t+1) = q_H(t)$$

(21)

the relative fitness of the construct:

$$w_{Total} = w_{Rec}w_{Dom} = 1$$

(22)

and the genetic load:

$$\mathcal{L}_{Total} = \frac{2q_H}{1 + q_H}$$

(23)

It is interesting to note that:

$$\mathcal{L}_{Total} \neq 1 - (1 - \mathcal{L}_{Rec})(1 - \mathcal{L}_{Dom})$$

(24)

These terms would be expected to be equal only if the load imposed by the two features acted independently. However, since the recessive costs of the construct act to remove construct homozygotes, the adult population is enriched for heterozygote carriers of the construct, in which the edits can be created, and the dominant effects can be inflicted. Thus, the dominant fitness costs affect a greater proportion of the population in the presence of the recessive cost than it would do if they acted upon a different locus.

1.3. Conditions for preventing drive.

Using equation 12 which describes the relative fitness (w) of the construct in terms of model parameters and the initial allele frequency of the construct, we next identify the parameter combinations with which w remains at or below that of the wildtype, and thus would not cause the construct to increase in frequency (drive). It can be shown that $w \leq 1$ if:

$$u \leq \frac{s_G - h_G s_G}{1 - h_G s_G} \tag{25}$$

If s_G = 1 this condition is necessarily satisfied, and thus if the genetic construct is inserted into a gene such that it causes fully penetrant recessive lethality the construct is not expected drive. Note that other parameter combinations will also prevent drive.

2. Simulations

We next extend the single-locus model to develop a series of more complex models that incorporate additional features including density-dependent population dynamics, sex-specificity and multiple loci.

2.1. Density dependent population dynamics

In all of the following models we incorporate population dynamics, tracking the density of males and females of each genotype independently and allowing for density dependent mortality between the hatchling and adult stage, i.e. as if larvae experience additional mortality when competing for resources. We also relax the assumption that the edit causes embryonic death and the construct causes pupal death, and instead allow either one to occur before or after density-dependent mortality. We also allow the option for fitness effects to manifest as reductions in fertility instead of survival. To summarise, in each generation males and females produce f fertilised eggs, assuming that the number of males in the population does not limit their production. Genotype-specific pre-density dependent mortality is applied at the embryo stage, before hatching. Density-dependent mortality is applied at the hatchling stage according to the Beverton-Holt model where the probability of surviving is $\theta \frac{\alpha}{\alpha+H}$, where θ is the density independent mortality probability the hatchling survives to pupation, α determines the strength of density dependent mortality and H is the number of hatchlings in the population. The intrinsic rate of increase (R_m) of a wild population before release is therefore $\frac{f\theta}{2}$. Since we report results in terms of numbers of females relative to pre-release, the precise value of α does not affect the results. Next genotype-specific post-density dependent mortality is applied to the pupae as they transition into adults. At this stage the adult population is censused. Where fitness costs affect fertility, only a portion of adults will be reproductively active. Genotype-specific costs which affect fertility are applied after censusing, assuming costs in females reduce the number of eggs they lay and costs in males reduce the probability the male will mate with a female. Note that by modelling fertility costs in this way we assume that fitness costs to males prevent them from mating (or they mate but females then mate again) and therefore the male costs do not impact the quantity of eggs laid by females.

2.2. A single-locus two-sex PDNE model

Our first extended model includes a single locus and 3 alleles (W, G and R), equivalent to the analytical model, but with two sexes. This involves tracking 6 male and 6 female genotypes.

Fitness effects

We allow the fitness costs to be sex-specific, where the superscript X represents the sex of the individuals (F = female, and M = male). We also relax the assumption of idealised dominance and penetrance of the dominant edit, incorporating an additional fitness cost and dominance coefficient for the edit (s_R^X and h_R^Xrespectively) and a fitness cost for heterozygotes for the construct and edit (s_{GR}^X).

The fitness of each of the genotypes relative to the wild-type is thus:

$$w_{WW}^X = 1$$
$$w_{RR}^X = (1 - s_R^X)$$
$$w_{GG}^X = (1 - s_G^X)$$
$$w_{WG}^X = (1 - s_G^X\, h_G^X)$$
$$w_{WR}^X = (1 - s_R^X h_R^X)$$
$$w_{GR}^X = (1 - s_{GR}^X)$$

(26)

where s_R^X, $s_G^X$and s_{GR}^X are the fitness costs in individuals homozygous for the edit, homozygous for the construct or heterozygous for the construct and edit respectively, and $h_R^X$and h_G^X are the dominance coefficients giving the proportion of homozygous costs felt in heterozygotes for the edit or construct when paired with a WT. This design also allowed us to model the fitness costs of a range of alternative strategies under a common framework (SI Appendix, Table S1). All fitness costs are assumed to cause lethality after density dependent mortality other than SIT, which causes death before.

Table S1 – The construct fitness parameters for a range of strategies including constructs that contain a dominant lethal allele that affects both sexes (SIT and RIDL) or only females (fsRIDL and fs-RIDL-drive /YLE), a sex-ratio distorter construct that has no fitness affects associated with its insertion locus (XS) and a protected dominant negative editor (PDNE) which causes

recessive lethality. Note that the other fitness parameters associated with the edit (s_R^X, h_R^X *and* s_{GR}^X) are zero for both sexes when modelling all strategies other than the PDNE and in the idealised PDNE case $s_R^F = s_R^M = s_{GR}^F = s_{GR}^M = 1$ when edits affect both sexes or $s_R^F = s_{GR}^F = 1$ and $s_R^M = s_{GR}^M = 0$ when the edit affects only females.

	s_G^F	s_G^M	h_G^F	h_G^M
SIT and RIDL	1	1	1	1
fsRIDL and fs-RIDL-drive/YLE	1	0	1	0
XS	0	0	0	0
PDNE	1	1	0	0

Gamete transmission

As in the analytical model, gamete transmission is altered in individuals heterozygous for the PDNE and WT alleles, since the WT is converted into an edit. Gamete transmission is also affected by the construct in the fs-RIDL-drive strategy (equivalent to a YLE when parameters are idealised) where in males heterozygous for the construct and WT, the WT is converted to the construct. To model both effects on gamete transmission we allow cleavage of the WT allele to occur in heterozygotes for the construct and WT with probability c, where $c = 1$ when idealised. The cleaved WT is then either converted to an edit with probability j or converted to a construct with probability $1 - j$, where $j = 1$ for an idealised PDNE and $j = 0$ for an idealised fs-RIDL-drive construct. To model the sex-ratio distortion effect of the XS, males who inherit the construct produce a proportion m of Y-bearing sperm, where $m = 1$ for an idealised XS construct and $m = 0.5$ otherwise.

2.3. Assessing evolutionary stability

We next extend the single locus two-sex PDNE model described in Section 2.2 to assess the impact of loss-of-function mutations on components of the genomic editor. After the release of a fully functional construct, we assume that each component mutates per generation with probability d, and allow for multiple components to acquire mutations within a single generation. This results in a total of 6 alleles and therefore 21 male and 21 female genotypes.

The genetic constructs carrying non-functional components inherit the recessive fitness costs associated with the insertion site being disrupted. For simplicity, and since we do not model evolutionary stability of an fs-RIDL-drive construct, we omit the j parameter, assuming the cleavage of the target site always results in the desired edit.

2.4. Assessing resistance alleles

We also extend the single locus two-sex PDNE model described in Section 2.2 to assess the impact of an editor which generates a portion of edits with recessive lethality. We now include two types of edits (R_D and R_R) representing edits with dominant or recessive fitness effects respectively, resulting in a total of 4 alleles and therefore 10 genotypes. In individuals carrying at least one genomic editor and one WT allele, the editor cleaves the WT allele with probability c, it is assumed that all cleaved alleles are converted to edits ($j = 1$) of which p are R_D edits and $1 - p$ are R_R edits. Since for simplicity we model this scenario using a single locus, editing only occurs in individuals heterozygous for the construct and WT, and therefore we do not model the case there homing of resistant alleles is possible.

2.5. Multi-locus two-sex model with recoded copy

We next modify the single locus two-sex PDNE model described in Section 2.2 to include a second locus, allowing us to model the case where the genomic editor targets a gene other than the one which it is inserted into. In this model we include two alleles at the first locus, the WT (W) and the genomic editor (G), and two alleles at the second, the WT target site susceptible to editing (W) and the edit (R). We allow for linkage between the loci, and therefore track 4 haplotypes, resulting in 10 male and 10 female genotypes.

Fitness effects

We again model the recessive fitness costs of the construct at the first locus using a fitness parameter (s_G^X) which describes the fitness cost in homozygotes that can differ depending on

sex, and a dominance coefficient (h_G^X) that describes the proportion of the costs in homozygotes that occur in heterozygotes. We model the dominant fitness costs of the edit at the second locus using the fitness parameter (s_R^X) and a dominance coefficient (h_R^X). The overall relative fitness of individuals is calculated by multiplying the relative fitness of the genotypes at each locus independently whilst taking into consideration the protective effects of the genomic editor on the edit, where a single editor protects against a single edit. For simplicity we assume no additional costs when there are more than two functional copies of a gene and that the genomic editor can fully restore function of an edit present in cis or trans (i.e. on either chromosome). Therefore the relative fitness of the 10 genotypes are:

$$w_{WW,WW}^X = 1$$

$$w_{WW,WR}^X(1 - s_R^X h_R^X)$$

$$w_{WR,WR}^X = (1 - s_R^X)$$

$$w_{WW,GW}^X = (1 - s_G^X\ h_G^X)$$

$$w_{WR,GW}^X = (1 - s_G^X\ h_G^X)$$

$$w_{WW,GR}^X = (1 - s_G^X\ h_G^X)$$

$$w_{WR,GR}^X = (1 - s_G^X\ h_G^X)(1 - s_R^X h_R^X)$$

$$w_{GW,GW}^X = (1 - s_G^X)$$

$$w_{GW,GR}^X = (1 - s_G^X)$$

$$w_{GR,GR}^X = (1 - s_G^X)$$

(27)

where, for example, $w_{WW,GR}^X$ indicates the fitness of an individual of sex X that is heterozygous for the genomic editor (G) and the wild-type (W) at the first locus and heterozygous for the edit (R) and the wild-type (W) at the second locus. The comma in the genotype label separates haplotypes, thus in this example, the G and R alleles would be located on the same chromosome (if there was linkage).

Gamete transmission

In individuals which carry at least one genomic editor and one WT target site, cleavage of each WT occurs with probability c, where $c = 1$ in the idealised case we model. Again we omit the j parameter, assuming the cleavage of the target site always results in the desired edit. We also allow linkage between the loci to vary, where recombination between two loci can occur with probability r. When the two loci are on different chromosomes $r = 0.5$ whereas when they are tightly linked $r = 0$.

2.6. Multi-locus two-sex model with an out of locus recoded copy

We next model the case where the genomic editor does not incorporate a recoded copy of the target site and therefore the construct is not protected against the edit but is instead released alongside a recoded or deleted version of the target site located *in situ*. Here the two-locus model described in Section 2.5 is extended to include a third allele (I) at the second locus which is both neutral and immune to editing by the editor. We again allow for linkage between the loci, including a total of 6 haplotypes and therefore 21 male and 21 female genotypes. Gamete transmission effects and recombination is modelled as described in Section 2.5

Fitness effects

Since there is no recoded version of the target site within the construct the fitness effects at the two loci act independently of one another. The relative fitness due to different combinations of alleles at first locus are:

$$w_{WW}^{X} = 1$$

$$w_{GG}^{X} = (1 - s_{G}^{X})$$

$$w_{WG}^{X} = (1 - s_{G}^{X}\, h_{G}^{X}) \qquad (28)$$

And at the second are:

$$w_{WW}^{X} = 1$$

$$w_{RR}^{X} = (1 - s_R^X)$$

$$w_{II}^{X} = (1 - s_I^X)$$

$$w_{WI}^{X} = (1 - s_I^X h_I^X)$$

$$w_{WR}^{X} = (1 - s_R^X h_R^X)$$

$$w_{IR}^{X} = (1 - s_{IR}^X)$$

(29)

where I refers to the allele which is immune to cleavage. s_I^X and s_{IR}^X are the fitness costs in individuals homozygous for the immune allele or heterozygous for the edit and immune allele respectively, and h_I^X is the dominance coefficients giving the proportion of homozygous costs felt in heterozygotes for the immune allele when paired with a WT.

The overall fitness of each genotype is then the product of the relative fitness due to each locus-specific genotype. For the example modelled in SI Appendix, Fig. S8. we assume the edit is created by knocking out a haplo-insufficient gene required for function in both sexes ($s_R^F = h_R^F = s_R^M = h_R^M = 1$), and the immune allele is a recoded version of this gene where homozygotes for the immune allele are fully fit ($s_I^X = h_I^X = 0$ and $s_{IR}^X = 1$), rather than a deletion of a HS^{ETDN} where they die or are sterile ($s_I^X = s_{IR}^X = 1$ and $h_I^X = 0$).

2.7. Incorporating a homing-based booster

To model use of a homing-based booster we extend the 2-locus model (with two alleles at each locus) described in Section 2.5 to incorporate a 3rd locus (with neutral fitness) which has two alleles, the wild-type (W) and the booster (B), resulting in 8 haplotypes and 36 male and 36 female genotypes. Homing of the genomic editor occurs in individuals heterozygous for the

editor and WT and carrying at least one copy of the booster. In these individuals cleavage of the WT occurs with probability c_H, after which the allele is converted to a genomic editor. Here we assume that the number of boosters makes no differences to homing efficiency and that it acts non-autonomously, i.e. has no impact on fitness or gamete transmission when present in the absence of the genomic editor. We assume that the three loci are arranged linearly, where the first is the insertion site of the booster, the second is the insertion site of the editor and third is the target site of the editor, and include two recombination rates, where $r1$ describes linkage between the first pair of loci and $r2$ described linkage between the second pair. When $r1 = r2 = 0.5$ all loci are located on separate chromosomes and alleles segregate independently. Note that when $r2 = 0$ and all model parameters are idealised, this is the equivalent of boosting a single locus PDNE design. For the idealised example we model in Fig. 5B, recessive costs affect both sexes ($s_G^F = s_G^M = 1$), the edit is female-specific ($s_R^F = 1$ and $s_R^M = 0$), editing and boosting are optimal ($c = c_H = 1$), the genomic editor and its own edit site are linked ($r2 = 0$) and the genomic editor and booster are unlinked ($r1 = 0.5$).

2.8. Incorporating a cleave and rescue booster

To model release of the construct alongside a cleave and rescue booster, we extend the single-locus model described in Section 2.2 (retaining all its features) to incorporate two additional loci, each with 2 alleles. At the 2nd locus there is the WT allele (W) and the booster (B), which results in of cleavage of the wild-type allele at the 3rd locus (W) to create an edited allele (T). This results in tracking of 12 haplotypes and therefore 78 male and 78 female genotypes.

Fitness effects

We model the fitness costs of the genomic editor and the edit it creates (within the same locus) in the same way as the model described in Section 2.2. We next incorporate an additional fitness cost (s_T^X) and dominance coefficient (h_T^X) to describe the fitness of the edit created by the booster. Since in this design the genomic editor is assumed to contain a rescue copy of the booster target site, the fitness of the individuals due to their genotype at the booster edit site depends on the number of copies of the genomic editor present elsewhere in the genome.

Thus, the following expressions give the fitness costs due to the booster-created edit in the absence of the PDNE:

$$w_{TT}^{X} = (1 - s_{T}^{X})$$
$$w_{WT}^{X} = (1 - s_{T}^{X} h_{T}^{X})$$
$$(30)$$

in the presence of one copy of the PDNE:

$$w_{TT}^{X} = (1 - s_{T}^{X} h_{T}^{X})$$
$$w_{WT}^{X} = 1$$
$$(31)$$

and in the presence of two copies of the PDNE:

$$w_{TT}^{X} = 1$$
$$w_{WT}^{X} = 1$$
$$(32)$$

where, for example, w_{WT}^{X} indicates an individual heterozygous for the wild-type and edit created by the booster construct, which can differ depending on the sex.

This parameterisation allows us to vary the fitness effects of the booster-created edit (recessive or dominant, and sex-specific or bisex) and ensure the rescue element in the PDNE functions in line with the fitness costs of the edit. For example, if the edit is recessive ($h_{T}^{X} = 0$) one functional copy of the target gene is sufficient (whether expressed from a PDNE or WT allele) whereas if the edit is dominant ($h_{T}^{X} = 1$), two functional copies of the rescue are needed for full fitness. Two copies of the PDNE are assumed sufficient to fully restore function in all genotypes and we assume no additional costs when there are more than two functional copies of the target gene. Again, the overall relative fitness of individuals is calculated by multiplying the relative fitness of the genotypes at each locus independently (taking into consideration the effects of rescue) and assuming there are no costs associated with the booster itself.

Gamete transmission

Changes to gamete transmission due to the PDNE are modelled similarly to the single-locus PDNE model, where the WT allele is cleaved with probability c, again assuming the cleavage of the target site always results in the creation of the desired edit. Additional changes occur if at least one booster is inherited alongside at least one WT target site of the booster, where each WT target allele is edited with probability c_T. To allow for linkage between the loci we assume they are all located in a chain along a single chromosome where the first locus contains the booster, the second the PDNE and the third the target site of the booster. We then allow recombination between each pair of loci to occur with probability $r1$ between the booster and PDNE and $r2$ between the PDNE and booster target site. When $r1 = r2 = 0.5$ all loci are located on separate chromosomes and alleles segregate independently.

For the idealised example we model in Fig. 5C, recessive costs affect both sexes ($s_G^F = s_G^M = 1$), the PDNE edit is dominant lethal that affects females ($s_R^F = h_R^F = 1$ and $s_R^M = h_R^M = 0$), the booster edit is recessive lethal that affects females ($s_T^F = 1$ and $s_T^M = h_T^M = h_T^F = 0$), editing by both the genomic editor and booster are optimal ($c = c_T = 1$), the PDNE and booster are partially linked ($r1 = 0.05$) and the PDNE and target site of the edit are unlinked ($r2 = 0.5$).

2.9. Calculating summary metrics from simulations

Post-simulation processing

The relative population densities were censused at the adult stage, after the application of genotype-specific fitness costs affecting survival but before those that affect fertility. Genotype frequencies were censused for each sex independently at the zygote stage, before genotype-specific lethality was applied, and allele frequencies were calculated from genotype frequencies averaged across both sexes. The correlation between the cleave and rescue booster and genomic editor was calculated as:

$$\frac{(p_{GB} - p_G * p_B)}{\sqrt{p_G(1-p_G)p_B(1-p_B)}} \qquad (33)$$

where, p_{GB} is the frequency of chromosomes containing both the genomic editor and cleave and rescue booster in the population and p_G and p_B are the frequency of the editor and booster in the population respectively.

Calculating release rate requirements

To calculate the release rate required for a strategy to reach a certain level of suppression, we began by simulating repeat releases of males at 0.1% (or 0.01% for boosted constructs) of the initial male population and evaluated the minimum relative number of females over the period of release. The release frequency was increased, and simulations were repeated until the population had been suppressed by at least the desired level, revealing the rate required to achieve the desired level of suppression within the desired time frame. Final release rates were all accurate to 3 decimal places (or 4 decimal places for boosted constructs).
